# Complete genome sequence for the thermoacidophilic archaeon *Sulfuracidifex* (f*. Sulfolobus*) *metallicus* DSM 6482

**DOI:** 10.1128/mra.00981-23

**Published:** 2023-12-06

**Authors:** Daniel J. Willard, Mohamad J. H. Manesh, Ryan G. Bing, Robert M. Kelly

**Affiliations:** 1 Department of Chemical and Biomolecular Engineering, North Carolina State University, Raleigh, North Carolina, USA; Portland State University, Portland, Oregon, USA

**Keywords:** thermoacidophile, archaea, crenarchaea, genome

## Abstract

Reported here is the complete genome sequence (2,191,724 bp) for the thermoacidophilic archaeon *Sulfuracidifex* (f*. Sulfolobus*) *metallicus* DSM 6482 (T_opt_ 65°C, pH_opt_ 2.0). This obligately chemolithoautotrophic microorganism is a prolific metal and sulfur oxidizer and has application in metal bioleaching operations. A multi-assembly reconciliation approach enabled closure of the genome.

## ANNOUNCEMENT

*Sulfuracidifex* (f*. Sulfolobus*) *metallicus* (DSM 6482, JCM 9184) was isolated from a solfataric field in Iceland in 1991 as an obligately chemolithoautotrophic aerobe and sulfur oxidizer that grows optimally at 65°C and pH 2.0 (1). Its classification was subsequently transferred to the genus *Sulfuracidifex* upon isolation of *Sulfuracidifex tepidarius*, based on closely related 16S rRNA gene sequences and physiological features (2); this re-assignment was supported further by detailed taxonomic and phylogenetic analysis (3). A draft genome assembly for *S. metallicus* DSM 6482 was first reported consisting 167 contigs (BioProject: PRJDB806), although later refined efforts resulted in a 5-contig assembly (4). Here, we report the closed genome sequence of *S. metallicus* DSM 6482, consisting of 2,194,724 bp containing 2,268 open reading frames.

The *S. metallicus* DSM 6482 was previously obtained from the Deutsche Sammlung von Mikroorganismen und Zellkuturen (DSMZ) and maintained in a freezer stock of 50:50 (vol%) glycerol:DSM88 medium (pH 4.5) at −80°C. *S. metallicus* cultures were grown at 65°C in modified DSM88 medium, per DSMZ recommendation. Genomic DNA was purified from liquid cultures using the NEB Monarch Genomic DNA Purification Kit (New England Biolabs, USA). DNA was barcoded and sequenced using Oxford Nanopore Technologies (UK) Native Barcoding Kit (SQK-NBD112-24) and R9.4.1 flow cell (FLO-MIN106D) on a MinION Mk1B without size selection. During barcoding and library preparation, DNA was quantified on a Qubit 4.0 with the 1× dsDNA HS Assay Kit (Invitrogen, Q33231). The multiplexed library was sequenced for 72 hours using MinKNOW v22.12.7 and Guppy v6.4.6 with live high-accuracy GPU base calling. MinionQC was used to verify the quality of the run (5).

**Fig 1:**
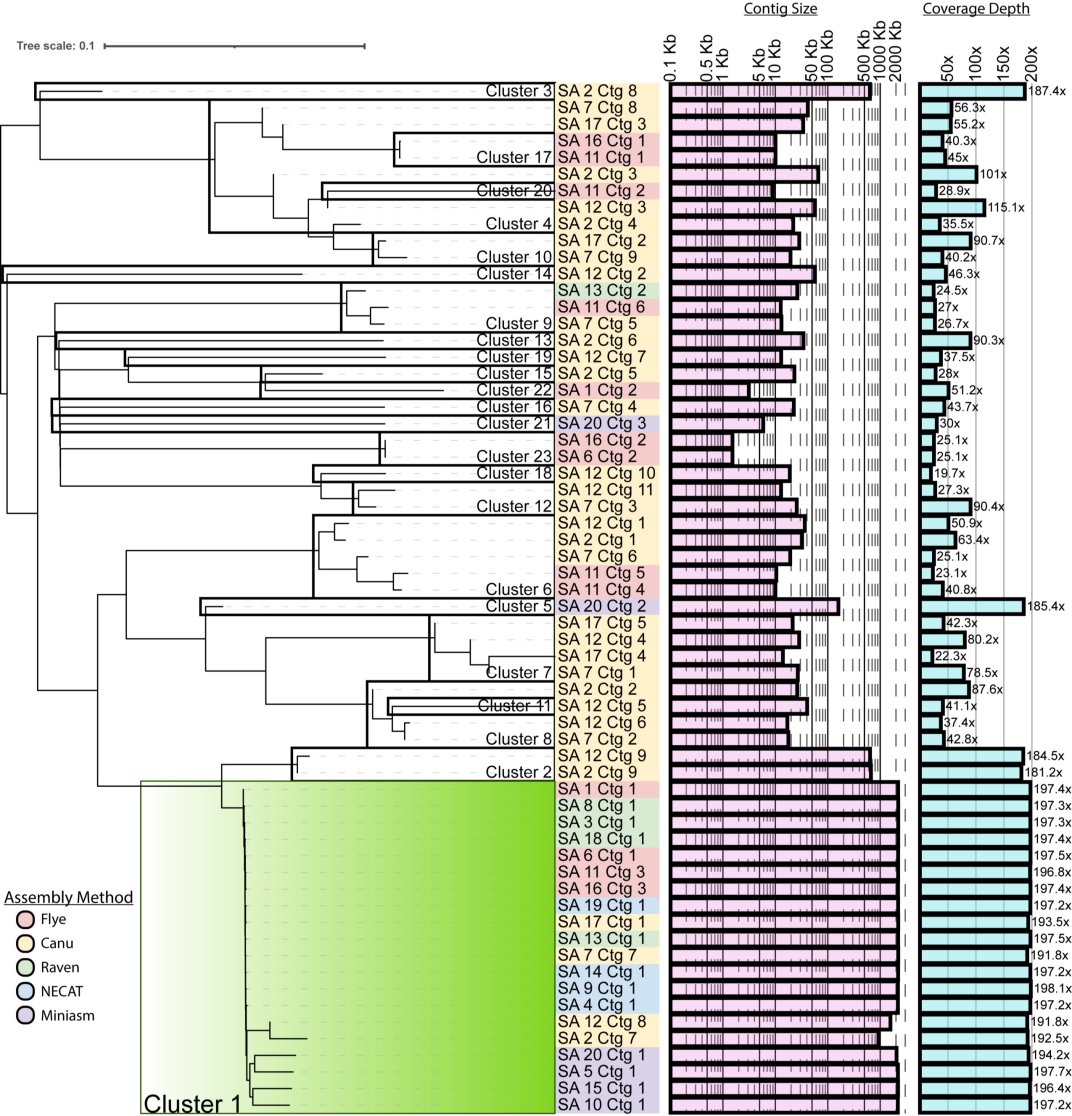
Hierarchical distance and clustering of sub-assembly (SA) contigs (Ctg). Contigs with a Mash distance <0.05 are considered within the same cluster. Contig length and read coverage depth are given for each contig leaf. Sub-assemblies are indicated by color: Flye (red), Canu (yellow), Raven (green), NECAT (blue), and Miniasm (purple). Contig Cluster 1 (highlighted in green) was selected for reconciliation of the genome.

Read trimming was performed by Guppy during base calling, and filtering was done with NanoFilt v2.8.0 using 1,000 bp length and Q10 quality cutoffs (6). The genome was assembled using the Trycycler v0.5.3 workflow (7), with sub-assemblies generated by Flye v2.9.1 (8), Canu v2.2 (9), Raven v1.6.1 (10), NECAT v0.0.1 (11), and Miniasm + Minipolish v0.3-r179/0.1.2 (12, 13). Briefly, reads were sub-sampled into 20 sets, and each assembler was used on 4 subsets to generate 20 sub-assemblies. Contigs were clustered using a Mash distance of 0.05 and visualized using the Interactive Tree of Life (14) (Fig. 1). With the exception of Cluster 1, the resulting clusters largely comprised contigs from only two to five sub-assemblies and exhibited erratic size and coverage, indicating these were likely misassembly artifacts. Therefore, only Cluster 1 was carried forward to reconcile and circularize the consensus genome sequence in Trycycler. The resulting circular genome was rotated using Circlator v1.5.5 (15) to start at the *dnaA* gene as predicted by Prodigal v0.5.2 (16). The assembly was polished with the full set of trimmed and filtered reads using Medaka v1.7.2 (https://github.com/nanoporetech/medaka), and further error correction was performed using Pilon v1.24 (17). Read mapping was performed with BWA v0.7.17 (18) and sorted and indexed with samtools v1.16.1 (19). The final assembly was evaluated with Quast v5.2.0 (20) for GC content and read coverage and CheckM v1.2.2 (21) for completeness and contamination (Table 1) and annotated using PGAP v2023-05-17.build6771 (22). Default settings were used for all software unless specified.

**TABLE 1:** Summary of assembly statistics

Genome accession ID	CP135238
BioProject	PRJNA1021186
BioSample	SAMN37544857
Sequence read archive	PRJNA1021186
No. of reads	120,335
Total reads	491,271,433 bp
Read n50	7,548 bp
Genome size	2,194,724 bp
GC content	38.57%
Read coverage	202×
Completeness	99.40%
Contamination	0.60%
No. of predicted genes	2,268

## Data Availability

The data for this whole-genome sequencing project have been deposited at NCBI and can be accessed using the accession numbers found in Table 1 for the BioProject, BioSample, and Genome. The raw reads used for the assembly are also accessible through NCBI using the SRA accession number provided in Table 1.
